# Forward–backward asymmetry of Drell–Yan lepton pairs in pp collisions at $$\sqrt{s} = 8$$$$\,\mathrm{TeV}$$

**DOI:** 10.1140/epjc/s10052-016-4156-z

**Published:** 2016-06-14

**Authors:** V. Khachatryan, A. M. Sirunyan, A. Tumasyan, W. Adam, E. Asilar, T. Bergauer, J. Brandstetter, E. Brondolin, M. Dragicevic, J. Erö, M. Flechl, M. Friedl, R. Frühwirth, V. M. Ghete, C. Hartl, N. Hörmann, J. Hrubec, M. Jeitler, V. Knünz, A. König, M. Krammer, I. Krätschmer, D. Liko, T. Matsushita, I. Mikulec, D. Rabady, B. Rahbaran, H. Rohringer, J. Schieck, R. Schöfbeck, J. Strauss, W. Treberer-Treberspurg, W. Waltenberger, C. -E. Wulz, V. Mossolov, N. Shumeiko, J. Suarez Gonzalez, S. Alderweireldt, T. Cornelis, E. A. De Wolf, X. Janssen, A. Knutsson, J. Lauwers, S. Luyckx, M. Van De Klundert, H. Van Haevermaet, P. Van Mechelen, N. Van Remortel, A. Van Spilbeeck, S. Abu Zeid, F. Blekman, J. D’Hondt, N. Daci, I. De Bruyn, K. Deroover, N. Heracleous, J. Keaveney, S. Lowette, L. Moreels, A. Olbrechts, Q. Python, D. Strom, S. Tavernier, W. Van Doninck, P. Van Mulders, G. P. Van Onsem, I. Van Parijs, P. Barria, H. Brun, C. Caillol, B. Clerbaux, G. De Lentdecker, G. Fasanella, L. Favart, A. Grebenyuk, G. Karapostoli, T. Lenzi, A. Léonard, T. Maerschalk, A. Marinov, L. Perniè, A. Randle-conde, T. Reis, T. Seva, C. Vander Velde, P. Vanlaer, R. Yonamine, F. Zenoni, F. Zhang, K. Beernaert, L. Benucci, A. Cimmino, S. Crucy, D. Dobur, A. Fagot, G. Garcia, M. Gul, J. Mccartin, A. A. Ocampo Rios, D. Poyraz, D. Ryckbosch, S. Salva, M. Sigamani, N. Strobbe, M. Tytgat, W. Van Driessche, E. Yazgan, N. Zaganidis, S. Basegmez, C. Beluffi, O. Bondu, S. Brochet, G. Bruno, A. Caudron, L. Ceard, G. G. Da Silveira, C. Delaere, D. Favart, L. Forthomme, A. Giammanco, J. Hollar, A. Jafari, P. Jez, M. Komm, V. Lemaitre, A. Mertens, M. Musich, C. Nuttens, L. Perrini, A. Pin, K. Piotrzkowski, A. Popov, L. Quertenmont, M. Selvaggi, M. Vidal Marono, N. Beliy, G. H. Hammad, W. L. Aldá Júnior, F. L. Alves, G. A. Alves, L. Brito, M. Correa Martins Junior, M. Hamer, C. Hensel, C. Mora Herrera, A. Moraes, M. E. Pol, P. Rebello Teles, E. Belchior Batista Das Chagas, W. Carvalho, J. Chinellato, A. Custódio, E. M. Da Costa, D. De Jesus Damiao, C. De Oliveira Martins, S. Fonseca De Souza, L. M. Huertas Guativa, H. Malbouisson, D. Matos Figueiredo, L. Mundim, H. Nogima, W. L. Prado Da Silva, A. Santoro, A. Sznajder, E. J. Tonelli Manganote, A. Vilela Pereira, S. Ahuja, C. A. Bernardes, A. De Souza Santos, S. Dogra, T. R. Fernandez Perez Tomei, E. M. Gregores, P. G. Mercadante, C. S. Moon, S. F. Novaes, Sandra S. Padula, D. Romero Abad, J. C. Ruiz Vargas, A. Aleksandrov, R. Hadjiiska, P. Iaydjiev, M. Rodozov, S. Stoykova, G. Sultanov, M. Vutova, A. Dimitrov, I. Glushkov, L. Litov, B. Pavlov, P. Petkov, M. Ahmad, J. G. Bian, G. M. Chen, H. S. Chen, M. Chen, T. Cheng, R. Du, C. H. Jiang, R. Plestina, F. Romeo, S. M. Shaheen, A. Spiezia, J. Tao, C. Wang, Z. Wang, H. Zhang, C. Asawatangtrakuldee, Y. Ban, Q. Li, S. Liu, Y. Mao, S. J. Qian, D. Wang, Z. Xu, C. Avila, A. Cabrera, L. F. Chaparro Sierra, C. Florez, J. P. Gomez, B. Gomez Moreno, J. C. Sanabria, N. Godinovic, D. Lelas, I. Puljak, P. M. Ribeiro Cipriano, Z. Antunovic, M. Kovac, V. Brigljevic, K. Kadija, J. Luetic, S. Micanovic, L. Sudic, A. Attikis, G. Mavromanolakis, J. Mousa, C. Nicolaou, F. Ptochos, P. A. Razis, H. Rykaczewski, M. Bodlak, M. Finger, M. Finger, Y. Assran, S. Elgammal, A. Ellithi Kamel, M. A. Mahmoud, Y. Mohammed, B. Calpas, M. Kadastik, M. Murumaa, M. Raidal, A. Tiko, C. Veelken, P. Eerola, J. Pekkanen, M. Voutilainen, J. Härkönen, V. Karimäki, R. Kinnunen, T. Lampén, K. Lassila-Perini, S. Lehti, T. Lindén, P. Luukka, T. Mäenpää, T. Peltola, E. Tuominen, J. Tuominiemi, E. Tuovinen, L. Wendland, J. Talvitie, T. Tuuva, M. Besancon, F. Couderc, M. Dejardin, D. Denegri, B. Fabbro, J. L. Faure, C. Favaro, F. Ferri, S. Ganjour, A. Givernaud, P. Gras, G. Hamel de Monchenault, P. Jarry, E. Locci, M. Machet, J. Malcles, J. Rander, A. Rosowsky, M. Titov, A. Zghiche, I. Antropov, S. Baffioni, F. Beaudette, P. Busson, L. Cadamuro, E. Chapon, C. Charlot, T. Dahms, O. Davignon, N. Filipovic, A. Florent, R. Granier de Cassagnac, S. Lisniak, L. Mastrolorenzo, P. Miné, I. N. Naranjo, M. Nguyen, C. Ochando, G. Ortona, P. Paganini, P. Pigard, S. Regnard, R. Salerno, J. B. Sauvan, Y. Sirois, T. Strebler, Y. Yilmaz, A. Zabi, J.-L. Agram, J. Andrea, A. Aubin, D. Bloch, J.-M. Brom, M. Buttignol, E. C. Chabert, N. Chanon, C. Collard, E. Conte, X. Coubez, J.-C. Fontaine, D. Gelé, U. Goerlach, C. Goetzmann, A.-C. Le Bihan, J. A. Merlin, K. Skovpen, P. Van Hove, S. Gadrat, S. Beauceron, C. Bernet, G. Boudoul, E. Bouvier, C. A. Carrillo Montoya, R. Chierici, D. Contardo, B. Courbon, P. Depasse, H. El Mamouni, J. Fan, J. Fay, S. Gascon, M. Gouzevitch, B. Ille, F. Lagarde, I. B. Laktineh, M. Lethuillier, L. Mirabito, A. L. Pequegnot, S. Perries, J. D. Ruiz Alvarez, D. Sabes, L. Sgandurra, V. Sordini, M. Vander Donckt, P. Verdier, S. Viret, T. Toriashvili, I. Bagaturia, C. Autermann, S. Beranek, M. Edelhoff, L. Feld, A. Heister, M. K. Kiesel, K. Klein, M. Lipinski, A. Ostapchuk, M. Preuten, F. Raupach, S. Schael, J. F. Schulte, T. Verlage, H. Weber, B. Wittmer, V. Zhukov, M. Ata, M. Brodski, E. Dietz-Laursonn, D. Duchardt, M. Endres, M. Erdmann, S. Erdweg, T. Esch, R. Fischer, A. Güth, T. Hebbeker, C. Heidemann, K. Hoepfner, D. Klingebiel, S. Knutzen, P. Kreuzer, M. Merschmeyer, A. Meyer, P. Millet, M. Olschewski, K. Padeken, P. Papacz, T. Pook, M. Radziej, H. Reithler, M. Rieger, F. Scheuch, L. Sonnenschein, D. Teyssier, S. Thüer, V. Cherepanov, Y. Erdogan, G. Flügge, H. Geenen, M. Geisler, F. Hoehle, B. Kargoll, T. Kress, Y. Kuessel, A. Künsken, J. Lingemann, A. Nehrkorn, A. Nowack, I. M. Nugent, C. Pistone, O. Pooth, A. Stahl, M. Aldaya Martin, I. Asin, N. Bartosik, O. Behnke, U. Behrens, A. J. Bell, K. Borras, A. Burgmeier, A. Cakir, A. Campbell, S. Choudhury, F. Costanza, C. Diez Pardos, G. Dolinska, S. Dooling, T. Dorland, G. Eckerlin, D. Eckstein, T. Eichhorn, G. Flucke, E. Gallo, J. Garay Garcia, A. Geiser, A. Gizhko, P. Gunnellini, J. Hauk, M. Hempel, H. Jung, A. Kalogeropoulos, O. Karacheban, M. Kasemann, P. Katsas, J. Kieseler, C. Kleinwort, I. Korol, W. Lange, J. Leonard, K. Lipka, A. Lobanov, W. Lohmann, R. Mankel, I. Marfin, I.-A. Melzer-Pellmann, A. B. Meyer, G. Mittag, J. Mnich, A. Mussgiller, S. Naumann-Emme, A. Nayak, E. Ntomari, H. Perrey, D. Pitzl, R. Placakyte, A. Raspereza, B. Roland, M. Ö. Sahin, P. Saxena, T. Schoerner-Sadenius, M. Schröder, C. Seitz, S. Spannagel, K. D. Trippkewitz, R. Walsh, C. Wissing, V. Blobel, M. Centis Vignali, A. R. Draeger, J. Erfle, E. Garutti, K. Goebel, D. Gonzalez, M. Görner, J. Haller, M. Hoffmann, R. S. Höing, A. Junkes, R. Klanner, R. Kogler, T. Lapsien, T. Lenz, I. Marchesini, D. Marconi, M. Meyer, D. Nowatschin, J. Ott, F. Pantaleo, T. Peiffer, A. Perieanu, N. Pietsch, J. Poehlsen, D. Rathjens, C. Sander, H. Schettler, P. Schleper, E. Schlieckau, A. Schmidt, J. Schwandt, V. Sola, H. Stadie, G. Steinbrück, H. Tholen, D. Troendle, E. Usai, L. Vanelderen, A. Vanhoefer, B. Vormwald, M. Akbiyik, C. Barth, C. Baus, J. Berger, C. Böser, E. Butz, T. Chwalek, F. Colombo, W. De Boer, A. Descroix, A. Dierlamm, S. Fink, F. Frensch, R. Friese, M. Giffels, A. Gilbert, D. Haitz, F. Hartmann, S. M. Heindl, U. Husemann, I. Katkov, A. Kornmayer, P. Lobelle Pardo, B. Maier, H. Mildner, M. U. Mozer, T. Müller, Th. Müller, M. Plagge, G. Quast, K. Rabbertz, S. Röcker, F. Roscher, G. Sieber, H. J. Simonis, F. M. Stober, R. Ulrich, J. Wagner-Kuhr, S. Wayand, M. Weber, T. Weiler, C. Wöhrmann, R. Wolf, G. Anagnostou, G. Daskalakis, T. Geralis, V. A. Giakoumopoulou, A. Kyriakis, D. Loukas, A. Psallidas, I. Topsis-Giotis, A. Agapitos, S. Kesisoglou, A. Panagiotou, N. Saoulidou, E. Tziaferi, I. Evangelou, G. Flouris, C. Foudas, P. Kokkas, N. Loukas, N. Manthos, I. Papadopoulos, E. Paradas, J. Strologas, G. Bencze, C. Hajdu, A. Hazi, P. Hidas, D. Horvath, F. Sikler, V. Veszpremi, G. Vesztergombi, A. J. Zsigmond, N. Beni, S. Czellar, J. Karancsi, J. Molnar, Z. Szillasi, M. Bartók, A. Makovec, P. Raics, Z. L. Trocsanyi, B. Ujvari, P. Mal, K. Mandal, D. K. Sahoo, N. Sahoo, S. K. Swain, S. Bansal, S. B. Beri, V. Bhatnagar, R. Chawla, R. Gupta, U. Bhawandeep, A. K. Kalsi, A. Kaur, M. Kaur, R. Kumar, A. Mehta, M. Mittal, J. B. Singh, G. Walia, Ashok Kumar, A. Bhardwaj, B. C. Choudhary, R. B. Garg, A. Kumar, S. Malhotra, M. Naimuddin, N. Nishu, K. Ranjan, R. Sharma, V. Sharma, S. Bhattacharya, K. Chatterjee, S. Dey, S. Dutta, Sa. Jain, N. Majumdar, A. Modak, K. Mondal, S. Mukherjee, S. Mukhopadhyay, A. Roy, D. Roy, S. Roy Chowdhury, S. Sarkar, M. Sharan, A. Abdulsalam, R. Chudasama, D. Dutta, V. Jha, V. Kumar, A. K. Mohanty, L. M. Pant, P. Shukla, A. Topkar, T. Aziz, S. Banerjee, S. Bhowmik, R. M. Chatterjee, R. K. Dewanjee, S. Dugad, S. Ganguly, S. Ghosh, M. Guchait, A. Gurtu, G. Kole, S. Kumar, B. Mahakud, M. Maity, G. Majumder, K. Mazumdar, S. Mitra, G. B. Mohanty, B. Parida, T. Sarkar, N. Sur, B. Sutar, N. Wickramage, S. Chauhan, S. Dube, S. Sharma, H. Bakhshiansohi, H. Behnamian, S. M. Etesami, A. Fahim, R. Goldouzian, M. Khakzad, M. Mohammadi Najafabadi, M. Naseri, S. Paktinat Mehdiabadi, F. Rezaei Hosseinabadi, B. Safarzadeh, M. Zeinali, M. Felcini, M. Grunewald, M. Abbrescia, C. Calabria, C. Caputo, A. Colaleo, D. Creanza, L. Cristella, N. De Filippis, M. De Palma, L. Fiore, G. Iaselli, G. Maggi, M. Maggi, G. Miniello, S. My, S. Nuzzo, A. Pompili, G. Pugliese, R. Radogna, A. Ranieri, G. Selvaggi, L. Silvestris, R. Venditti, P. Verwilligen, G. Abbiendi, C. Battilana, A. C. Benvenuti, D. Bonacorsi, S. Braibant-Giacomelli, L. Brigliadori, R. Campanini, P. Capiluppi, A. Castro, F. R. Cavallo, S. S. Chhibra, G. Codispoti, M. Cuffiani, G. M. Dallavalle, F. Fabbri, A. Fanfani, D. Fasanella, P. Giacomelli, C. Grandi, L. Guiducci, S. Marcellini, G. Masetti, A. Montanari, F. L. Navarria, A. Perrotta, A. M. Rossi, T. Rovelli, G. P. Siroli, N. Tosi, R. Travaglini, G. Cappello, M. Chiorboli, S. Costa, F. Giordano, R. Potenza, A. Tricomi, C. Tuve, G. Barbagli, V. Ciulli, C. Civinini, R. D’Alessandro, E. Focardi, S. Gonzi, V. Gori, P. Lenzi, M. Meschini, S. Paoletti, G. Sguazzoni, A. Tropiano, L. Viliani, L. Benussi, S. Bianco, F. Fabbri, D. Piccolo, F. Primavera, V. Calvelli, F. Ferro, M. Lo Vetere, M. R. Monge, E. Robutti, S. Tosi, L. Brianza, M. E. Dinardo, S. Fiorendi, S. Gennai, R. Gerosa, A. Ghezzi, P. Govoni, S. Malvezzi, R. A. Manzoni, B. Marzocchi, D. Menasce, L. Moroni, M. Paganoni, D. Pedrini, S. Ragazzi, N. Redaelli, T. Tabarelli de Fatis, S. Buontempo, N. Cavallo, S. Di Guida, M. Esposito, F. Fabozzi, A. O. M. Iorio, G. Lanza, L. Lista, S. Meola, M. Merola, P. Paolucci, C. Sciacca, F. Thyssen, P. Azzi, N. Bacchetta, L. Benato, D. Bisello, A. Boletti, R. Carlin, P. Checchia, M. Dall’Osso, T. Dorigo, S. Fantinel, F. Fanzago, F. Gasparini, U. Gasparini, F. Gonella, A. Gozzelino, S. Lacaprara, M. Margoni, A. T. Meneguzzo, F. Montecassiano, J. Pazzini, N. Pozzobon, P. Ronchese, F. Simonetto, E. Torassa, M. Tosi, M. Zanetti, P. Zotto, A. Zucchetta, G. Zumerle, A. Braghieri, A. Magnani, P. Montagna, S. P. Ratti, V. Re, C. Riccardi, P. Salvini, I. Vai, P. Vitulo, L. Alunni Solestizi, M. Biasini, G. M. Bilei, D. Ciangottini, L. Fanò, P. Lariccia, G. Mantovani, M. Menichelli, A. Saha, A. Santocchia, K. Androsov, P. Azzurri, G. Bagliesi, J. Bernardini, T. Boccali, R. Castaldi, M. A. Ciocci, R. Dell’Orso, S. Donato, G. Fedi, L. Foà, A. Giassi, M. T. Grippo, F. Ligabue, T. Lomtadze, L. Martini, A. Messineo, F. Palla, A. Rizzi, A. Savoy-Navarro, A. T. Serban, P. Spagnolo, R. Tenchini, G. Tonelli, A. Venturi, P. G. Verdini, L. Barone, F. Cavallari, G. D’imperio, D. Del Re, M. Diemoz, S. Gelli, C. Jorda, E. Longo, F. Margaroli, P. Meridiani, G. Organtini, R. Paramatti, F. Preiato, S. Rahatlou, C. Rovelli, F. Santanastasio, P. Traczyk, N. Amapane, R. Arcidiacono, S. Argiro, M. Arneodo, R. Bellan, C. Biino, N. Cartiglia, M. Costa, R. Covarelli, A. Degano, N. Demaria, L. Finco, B. Kiani, C. Mariotti, S. Maselli, E. Migliore, V. Monaco, E. Monteil, M. M. Obertino, L. Pacher, N. Pastrone, M. Pelliccioni, G. L. Pinna Angioni, F. Ravera, A. Romero, M. Ruspa, R. Sacchi, A. Solano, A. Staiano, U. Tamponi, S. Belforte, V. Candelise, M. Casarsa, F. Cossutti, G. Della Ricca, B. Gobbo, C. La Licata, M. Marone, A. Schizzi, A. Zanetti, A. Kropivnitskaya, S. K. Nam, D. H. Kim, G. N. Kim, M. S. Kim, D. J. Kong, S. Lee, Y. D. Oh, A. Sakharov, D. C. Son, J. A. Brochero Cifuentes, H. Kim, T. J. Kim, S. Song, S. Choi, Y. Go, D. Gyun, B. Hong, M. Jo, H. Kim, Y. Kim, B. Lee, K. Lee, K. S. Lee, S. Lee, S. K. Park, Y. Roh, H. D. Yoo, M. Choi, H. Kim, J. H. Kim, J. S. H. Lee, I. C. Park, G. Ryu, M. S. Ryu, Y. Choi, J. Goh, D. Kim, E. Kwon, J. Lee, I. Yu, A. Juodagalvis, J. Vaitkus, I. Ahmed, Z. A. Ibrahim, J. R. Komaragiri, M. A. B. Md Ali, F. Mohamad Idris, W. A. T. Wan Abdullah, M. N. Yusli, E. Casimiro Linares, H. Castilla-Valdez, E. De La Cruz-Burelo, I. Heredia-De La Cruz, A. Hernandez-Almada, R. Lopez-Fernandez, A. Sanchez-Hernandez, S. Carrillo Moreno, F. Vazquez Valencia, I. Pedraza, H. A. Salazar Ibarguen, A. Morelos Pineda, D. Krofcheck, P. H. Butler, A. Ahmad, M. Ahmad, Q. Hassan, H. R. Hoorani, W. A. Khan, T. Khurshid, M. Shoaib, H. Bialkowska, M. Bluj, B. Boimska, T. Frueboes, M. Górski, M. Kazana, K. Nawrocki, K. Romanowska-Rybinska, M. Szleper, P. Zalewski, G. Brona, K. Bunkowski, A. Byszuk, K. Doroba, A. Kalinowski, M. Konecki, J. Krolikowski, M. Misiura, M. Olszewski, M. Walczak, P. Bargassa, C. Beirão Da Cruz E Silva, A. Di Francesco, P. Faccioli, P. G. Ferreira Parracho, M. Gallinaro, N. Leonardo, L. Lloret Iglesias, F. Nguyen, J. Rodrigues Antunes, J. Seixas, O. Toldaiev, D. Vadruccio, J. Varela, P. Vischia, S. Afanasiev, P. Bunin, M. Gavrilenko, I. Golutvin, I. Gorbunov, A. Kamenev, V. Karjavin, V. Konoplyanikov, A. Lanev, A. Malakhov, V. Matveev, P. Moisenz, V. Palichik, V. Perelygin, S. Shmatov, S. Shulha, N. Skatchkov, V. Smirnov, A. Zarubin, V. Golovtsov, Y. Ivanov, V. Kim, E. Kuznetsova, P. Levchenko, V. Murzin, V. Oreshkin, I. Smirnov, V. Sulimov, L. Uvarov, S. Vavilov, A. Vorobyev, Yu. Andreev, A. Dermenev, S. Gninenko, N. Golubev, A. Karneyeu, M. Kirsanov, N. Krasnikov, A. Pashenkov, D. Tlisov, A. Toropin, V. Epshteyn, V. Gavrilov, N. Lychkovskaya, V. Popov, l. Pozdnyakov, G. Safronov, A. Spiridonov, E. Vlasov, A. Zhokin, A. Bylinkin, V. Andreev, M. Azarkin, I. Dremin, M. Kirakosyan, A. Leonidov, G. Mesyats, S. V. Rusakov, A. Baskakov, A. Belyaev, E. Boos, M. Dubinin, L. Dudko, A. Ershov, A. Gribushin, V. Klyukhin, O. Kodolova, I. Lokhtin, I. Myagkov, S. Obraztsov, S. Petrushanko, V. Savrin, A Snigirev, I. Azhgirey, I. Bayshev, S. Bitioukov, V. Kachanov, A. Kalinin, D. Konstantinov, V. Krychkine, V. Petrov, R. Ryutin, A. Sobol, L. Tourtchanovitch, S. Troshin, N. Tyurin, A. Uzunian, A. Volkov, P. Adzic, J. Milosevic, V. Rekovic, J. Alcaraz Maestre, E. Calvo, M. Cerrada, M. Chamizo Llatas, N. Colino, B. De La Cruz, A. Delgado Peris, D. Domínguez Vázquez, A. Escalante Del Valle, C. Fernandez Bedoya, J. P. Fernández Ramos, J. Flix, M. C. Fouz, P. Garcia-Abia, O. Gonzalez Lopez, S. Goy Lopez, J. M. Hernandez, M. I. Josa, E. Navarro De Martino, A. Pérez-Calero Yzquierdo, J. Puerta Pelayo, A. Quintario Olmeda, I. Redondo, L. Romero, J. Santaolalla, M. S. Soares, C. Albajar, J. F. de Trocóniz, M. Missiroli, D. Moran, J. Cuevas, J. Fernandez Menendez, S. Folgueras, I. Gonzalez Caballero, E. Palencia Cortezon, J. M. Vizan Garcia, I. J. Cabrillo, A. Calderon, J. R. Castiñeiras De Saa, P. De Castro Manzano, J. Duarte Campderros, M. Fernandez, J. Garcia-Ferrero, G. Gomez, A. Lopez Virto, J. Marco, R. Marco, C. Martinez Rivero, F. Matorras, F. J. Munoz Sanchez, J. Piedra Gomez, T. Rodrigo, A. Y. Rodríguez-Marrero, A. Ruiz-Jimeno, L. Scodellaro, N. Trevisani, I. Vila, R. Vilar Cortabitarte, D. Abbaneo, E. Auffray, G. Auzinger, M. Bachtis, P. Baillon, A. H. Ball, D. Barney, A. Benaglia, J. Bendavid, L. Benhabib, J. F. Benitez, G. M. Berruti, P. Bloch, A. Bocci, A. Bonato, C. Botta, H. Breuker, T. Camporesi, R. Castello, G. Cerminara, M. D’Alfonso, D. d’Enterria, A. Dabrowski, V. Daponte, A. David, M. De Gruttola, F. De Guio, A. De Roeck, S. De Visscher, E. Di Marco, M. Dobson, M. Dordevic, B. Dorney, T. du Pree, M. Dünser, N. Dupont, A. Elliott-Peisert, G. Franzoni, W. Funk, D. Gigi, K. Gill, D. Giordano, M. Girone, F. Glege, R. Guida, S. Gundacker, M. Guthoff, J. Hammer, P. Harris, J. Hegeman, V. Innocente, P. Janot, H. Kirschenmann, M. J. Kortelainen, K. Kousouris, K. Krajczar, P. Lecoq, C. Lourenço, M. T. Lucchini, N. Magini, L. Malgeri, M. Mannelli, A. Martelli, L. Masetti, F. Meijers, S. Mersi, E. Meschi, F. Moortgat, S. Morovic, M. Mulders, M. V. Nemallapudi, H. Neugebauer, S. Orfanelli, L. Orsini, L. Pape, E. Perez, M. Peruzzi, A. Petrilli, G. Petrucciani, A. Pfeiffer, D. Piparo, A. Racz, G. Rolandi, M. Rovere, M. Ruan, H. Sakulin, C. Schäfer, C. Schwick, M. Seidel, A. Sharma, P. Silva, M. Simon, P. Sphicas, J. Steggemann, B. Stieger, M. Stoye, Y. Takahashi, D. Treille, A. Triossi, A. Tsirou, G. I. Veres, N. Wardle, H. K. Wöhri, A. Zagozdzinska, W. D. Zeuner, W. Bertl, K. Deiters, W. Erdmann, R. Horisberger, Q. Ingram, H. C. Kaestli, D. Kotlinski, U. Langenegger, D. Renker, T. Rohe, F. Bachmair, L. Bäni, L. Bianchini, B. Casal, G. Dissertori, M. Dittmar, M. Donegà, P. Eller, C. Grab, C. Heidegger, D. Hits, J. Hoss, G. Kasieczka, W. Lustermann, B. Mangano, M. Marionneau, P. Martinez Ruiz del Arbol, M. Masciovecchio, D. Meister, F. Micheli, P. Musella, F. Nessi-Tedaldi, F. Pandolfi, J. Pata, F. Pauss, L. Perrozzi, M. Quittnat, M. Rossini, A. Starodumov, M. Takahashi, V. R. Tavolaro, K. Theofilatos, R. Wallny, T. K. Aarrestad, C. Amsler, L. Caminada, M. F. Canelli, V. Chiochia, A. De Cosa, C. Galloni, A. Hinzmann, T. Hreus, B. Kilminster, C. Lange, J. Ngadiuba, D. Pinna, P. Robmann, F. J. Ronga, D. Salerno, Y. Yang, M. Cardaci, K. H. Chen, T. H. Doan, Sh. Jain, R. Khurana, M. Konyushikhin, C. M. Kuo, W. Lin, Y. J. Lu, S. S. Yu, Arun Kumar, R. Bartek, P. Chang, Y. H. Chang, Y. W. Chang, Y. Chao, K. F. Chen, P. H. Chen, C. Dietz, F. Fiori, U. Grundler, W.-S. Hou, Y. Hsiung, Y. F. Liu, R.-S. Lu, M. Miñano Moya, E. Petrakou, J. f. Tsai, Y. M. Tzeng, B. Asavapibhop, K. Kovitanggoon, G. Singh, N. Srimanobhas, N. Suwonjandee, A. Adiguzel, M. N. Bakirci, S. Cerci, Z. S. Demiroglu, C. Dozen, I. Dumanoglu, E. Eskut, S. Girgis, G. Gokbulut, Y. Guler, E. Gurpinar, I. Hos, E. E. Kangal, A. Kayis Topaksu, G. Onengut, K. Ozdemir, A. Polatoz, M. Vergili, C. Zorbilmez, I. V. Akin, B. Bilin, S. Bilmis, B. Isildak, G. Karapinar, M. Yalvac, M. Zeyrek, E. Gülmez, M. Kaya, O. Kaya, E. A. Yetkin, T. Yetkin, K. Cankocak, S. Sen, F. I. Vardarlı, B. Grynyov, L. Levchuk, P. Sorokin, R. Aggleton, F. Ball, L. Beck, J. J. Brooke, E. Clement, D. Cussans, H. Flacher, J. Goldstein, M. Grimes, G. P. Heath, H. F. Heath, J. Jacob, L. Kreczko, C. Lucas, Z. Meng, D. M. Newbold, S. Paramesvaran, A. Poll, T. Sakuma, S. Seif El Nasr-storey, S. Senkin, D. Smith, V. J. Smith, K. W. Bell, A. Belyaev, C. Brew, R. M. Brown, L. Calligaris, D. Cieri, D. J. A. Cockerill, J. A. Coughlan, K. Harder, S. Harper, E. Olaiya, D. Petyt, C. H. Shepherd-Themistocleous, A. Thea, I. R. Tomalin, T. Williams, W. J. Womersley, S. D. Worm, M. Baber, R. Bainbridge, O. Buchmuller, A. Bundock, D. Burton, S. Casasso, M. Citron, D. Colling, L. Corpe, N. Cripps, P. Dauncey, G. Davies, A. De Wit, M. Della Negra, P. Dunne, A. Elwood, W. Ferguson, J. Fulcher, D. Futyan, G. Hall, G. Iles, M. Kenzie, R. Lane, R. Lucas, L. Lyons, A.-M. Magnan, S. Malik, J. Nash, A. Nikitenko, J. Pela, M. Pesaresi, K. Petridis, D. M. Raymond, A. Richards, A. Rose, C. Seez, A. Tapper, K. Uchida, M. Vazquez Acosta, T. Virdee, S. C. Zenz, J. E. Cole, P. R. Hobson, A. Khan, P. Kyberd, D. Leggat, D. Leslie, I. D. Reid, P. Symonds, L. Teodorescu, M. Turner, A. Borzou, K. Call, J. Dittmann, K. Hatakeyama, H. Liu, N. Pastika, O. Charaf, S. I. Cooper, C. Henderson, P. Rumerio, D. Arcaro, A. Avetisyan, T. Bose, C. Fantasia, D. Gastler, P. Lawson, D. Rankin, C. Richardson, J. Rohlf, J. St. John, L. Sulak, D. Zou, J. Alimena, E. Berry, S. Bhattacharya, D. Cutts, N. Dhingra, A. Ferapontov, A. Garabedian, J. Hakala, U. Heintz, E. Laird, G. Landsberg, Z. Mao, M. Narain, S. Piperov, S. Sagir, R. Syarif, R. Breedon, G. Breto, M. Calderon De La Barca Sanchez, S. Chauhan, M. Chertok, J. Conway, R. Conway, P. T. Cox, R. Erbacher, M. Gardner, W. Ko, R. Lander, M. Mulhearn, D. Pellett, J. Pilot, F. Ricci-Tam, S. Shalhout, J. Smith, M. Squires, D. Stolp, M. Tripathi, S. Wilbur, R. Yohay, R. Cousins, P. Everaerts, C. Farrell, J. Hauser, M. Ignatenko, D. Saltzberg, E. Takasugi, V. Valuev, M. Weber, K. Burt, R. Clare, J. Ellison, J. W. Gary, G. Hanson, J. Heilman, M. Ivova PANEVA, P. Jandir, E. Kennedy, F. Lacroix, O. R. Long, A. Luthra, M. Malberti, M. Olmedo Negrete, A. Shrinivas, H. Wei, S. Wimpenny, B. R. Yates, J. G. Branson, G. B. Cerati, S. Cittolin, R. T. D’Agnolo, M. Derdzinski, A. Holzner, R. Kelley, D. Klein, J. Letts, I. Macneill, D. Olivito, S. Padhi, M. Pieri, M. Sani, V. Sharma, S. Simon, M. Tadel, A. Vartak, S. Wasserbaech, C. Welke, F. Würthwein, A. Yagil, G. Zevi Della Porta, J. Bradmiller-Feld, C. Campagnari, A. Dishaw, V. Dutta, K. Flowers, M. Franco Sevilla, P. Geffert, C. George, F. Golf, L. Gouskos, J. Gran, J. Incandela, N. Mccoll, S. D. Mullin, J. Richman, D. Stuart, I. Suarez, C. West, J. Yoo, D. Anderson, A. Apresyan, A. Bornheim, J. Bunn, Y. Chen, J. Duarte, A. Mott, H. B. Newman, C. Pena, M. Pierini, M. Spiropulu, J. R. Vlimant, S. Xie, R. Y. Zhu, M. B. Andrews, V. Azzolini, A. Calamba, B. Carlson, T. Ferguson, M. Paulini, J. Russ, M. Sun, H. Vogel, I. Vorobiev, J. P. Cumalat, W. T. Ford, A. Gaz, F. Jensen, A. Johnson, M. Krohn, T. Mulholland, U. Nauenberg, K. Stenson, S. R. Wagner, J. Alexander, A. Chatterjee, J. Chaves, J. Chu, S. Dittmer, N. Eggert, N. Mirman, G. Nicolas Kaufman, J. R. Patterson, A. Rinkevicius, A. Ryd, L. Skinnari, L. Soffi, W. Sun, S. M. Tan, W. D. Teo, J. Thom, J. Thompson, J. Tucker, Y. Weng, P. Wittich, S. Abdullin, M. Albrow, J. Anderson, G. Apollinari, S. Banerjee, L. A. T. Bauerdick, A. Beretvas, J. Berryhill, P. C. Bhat, G. Bolla, K. Burkett, J. N. Butler, H. W. K. Cheung, F. Chlebana, S. Cihangir, V. D. Elvira, I. Fisk, J. Freeman, E. Gottschalk, L. Gray, D. Green, S. Grünendahl, O. Gutsche, J. Hanlon, D. Hare, R. M. Harris, S. Hasegawa, J. Hirschauer, Z. Hu, S. Jindariani, M. Johnson, U. Joshi, A. W. Jung, B. Klima, B. Kreis, S. Kwan, S. Lammel, J. Linacre, D. Lincoln, R. Lipton, T. Liu, R. Lopes De Sá, J. Lykken, K. Maeshima, J. M. Marraffino, V. I. Martinez Outschoorn, S. Maruyama, D. Mason, P. McBride, P. Merkel, K. Mishra, S. Mrenna, S. Nahn, C. Newman-Holmes, V. O’Dell, K. Pedro, O. Prokofyev, G. Rakness, E. Sexton-Kennedy, A. Soha, W. J. Spalding, L. Spiegel, L. Taylor, S. Tkaczyk, N. V. Tran, L. Uplegger, E. W. Vaandering, C. Vernieri, M. Verzocchi, R. Vidal, H. A. Weber, A. Whitbeck, F. Yang, D. Acosta, P. Avery, P. Bortignon, D. Bourilkov, A. Carnes, M. Carver, D. Curry, S. Das, G. P. Di Giovanni, R. D. Field, I. K. Furic, S. V. Gleyzer, J. Hugon, J. Konigsberg, A. Korytov, J. F. Low, P. Ma, K. Matchev, H. Mei, P. Milenovic, G. Mitselmakher, D. Rank, R. Rossin, L. Shchutska, M. Snowball, D. Sperka, N. Terentyev, L. Thomas, J. Wang, S. Wang, J. Yelton, S. Hewamanage, S. Linn, P. Markowitz, G. Martinez, J. L. Rodriguez, A. Ackert, J. R. Adams, T. Adams, A. Askew, J. Bochenek, B. Diamond, J. Haas, S. Hagopian, V. Hagopian, K. F. Johnson, A. Khatiwada, H. Prosper, M. Weinberg, M. M. Baarmand, V. Bhopatkar, S. Colafranceschi, M. Hohlmann, H. Kalakhety, D. Noonan, T. Roy, F. Yumiceva, M. R. Adams, L. Apanasevich, D. Berry, R. R. Betts, I. Bucinskaite, R. Cavanaugh, O. Evdokimov, L. Gauthier, C. E. Gerber, D. J. Hofman, P. Kurt, C. O’Brien, l. D. Sandoval Gonzalez, C. Silkworth, P. Turner, N. Varelas, Z. Wu, M. Zakaria, B. Bilki, W. Clarida, K. Dilsiz, S. Durgut, R. P. Gandrajula, M. Haytmyradov, V. Khristenko, J.-P. Merlo, H. Mermerkaya, A. Mestvirishvili, A. Moeller, J. Nachtman, H. Ogul, Y. Onel, F. Ozok, A. Penzo, C. Snyder, E. Tiras, J. Wetzel, K. Yi, I. Anderson, B. A. Barnett, B. Blumenfeld, N. Eminizer, D. Fehling, L. Feng, A. V. Gritsan, P. Maksimovic, C. Martin, M. Osherson, J. Roskes, A. Sady, U. Sarica, M. Swartz, M. Xiao, Y. Xin, C. You, P. Baringer, A. Bean, G. Benelli, C. Bruner, R. P. Kenny, D. Majumder, M. Malek, M. Murray, S. Sanders, R. Stringer, Q. Wang, A. Ivanov, K. Kaadze, S. Khalil, M. Makouski, Y. Maravin, A. Mohammadi, L. K. Saini, N. Skhirtladze, S. Toda, D. Lange, F. Rebassoo, D. Wright, C. Anelli, A. Baden, O. Baron, A. Belloni, B. Calvert, S. C. Eno, C. Ferraioli, J. A. Gomez, N. J. Hadley, S. Jabeen, R. G. Kellogg, T. Kolberg, J. Kunkle, Y. Lu, A. C. Mignerey, Y. H. Shin, A. Skuja, M. B. Tonjes, S. C. Tonwar, A. Apyan, R. Barbieri, A. Baty, K. Bierwagen, S. Brandt, W. Busza, I. A. Cali, Z. Demiragli, L. Di Matteo, G. Gomez Ceballos, M. Goncharov, D. Gulhan, Y. Iiyama, G. M. Innocenti, M. Klute, D. Kovalskyi, Y. S. Lai, Y.-J. Lee, A. Levin, P. D. Luckey, A. C. Marini, C. Mcginn, C. Mironov, S. Narayanan, X. Niu, C. Paus, D. Ralph, C. Roland, G. Roland, J. Salfeld-Nebgen, G. S. F. Stephans, K. Sumorok, M. Varma, D. Velicanu, J. Veverka, J. Wang, T. W. Wang, B. Wyslouch, M. Yang, V. Zhukova, B. Dahmes, A. Evans, A. Finkel, A. Gude, P. Hansen, S. Kalafut, S. C. Kao, K. Klapoetke, Y. Kubota, Z. Lesko, J. Mans, S. Nourbakhsh, N. Ruckstuhl, R. Rusack, N. Tambe, J. Turkewitz, J. G. Acosta, S. Oliveros, E. Avdeeva, K. Bloom, S. Bose, D. R. Claes, A. Dominguez, C. Fangmeier, R. Gonzalez Suarez, R. Kamalieddin, J. Keller, D. Knowlton, I. Kravchenko, J. Lazo-Flores, F. Meier, J. Monroy, F. Ratnikov, J. E. Siado, G. R. Snow, M. Alyari, J. Dolen, J. George, A. Godshalk, C. Harrington, I. Iashvili, J. Kaisen, A. Kharchilava, A. Kumar, S. Rappoccio, B. Roozbahani, G. Alverson, E. Barberis, D. Baumgartel, M. Chasco, A. Hortiangtham, A. Massironi, D. M. Morse, D. Nash, T. Orimoto, R. Teixeira De Lima, D. Trocino, R.-J. Wang, D. Wood, J. Zhang, K. A. Hahn, A. Kubik, N. Mucia, N. Odell, B. Pollack, A. Pozdnyakov, M. Schmitt, S. Stoynev, K. Sung, M. Trovato, M. Velasco, A. Brinkerhoff, N. Dev, M. Hildreth, C. Jessop, D. J. Karmgard, N. Kellams, K. Lannon, S. Lynch, N. Marinelli, F. Meng, C. Mueller, Y. Musienko, T. Pearson, M. Planer, A. Reinsvold, R. Ruchti, G. Smith, S. Taroni, N. Valls, M. Wayne, M. Wolf, A. Woodard, L. Antonelli, J. Brinson, B. Bylsma, L. S. Durkin, S. Flowers, A. Hart, C. Hill, R. Hughes, W. Ji, K. Kotov, T. Y. Ling, B. Liu, W. Luo, D. Puigh, M. Rodenburg, B. L. Winer, H. W. Wulsin, O. Driga, P. Elmer, J. Hardenbrook, P. Hebda, S. A. Koay, P. Lujan, D. Marlow, T. Medvedeva, M. Mooney, J. Olsen, C. Palmer, P. Piroué, X. Quan, H. Saka, D. Stickland, C. Tully, J. S. Werner, A. Zuranski, S. Malik, V. E. Barnes, D. Benedetti, D. Bortoletto, L. Gutay, M. K. Jha, M. Jones, K. Jung, D. H. Miller, N. Neumeister, B. C. Radburn-Smith, X. Shi, I. Shipsey, D. Silvers, J. Sun, A. Svyatkovskiy, F. Wang, W. Xie, L. Xu, N. Parashar, J. Stupak, A. Adair, B. Akgun, Z. Chen, K. M. Ecklund, F. J. M. Geurts, M. Guilbaud, W. Li, B. Michlin, M. Northup, B. P. Padley, R. Redjimi, J. Roberts, J. Rorie, Z. Tu, J. Zabel, B. Betchart, A. Bodek, P. de Barbaro, R. Demina, Y. Eshaq, T. Ferbel, M. Galanti, A. Garcia-Bellido, J. Han, A. Harel, O. Hindrichs, A. Khukhunaishvili, G. Petrillo, P. Tan, M. Verzetti, S. Arora, A. Barker, J. P. Chou, C. Contreras-Campana, E. Contreras-Campana, D. Duggan, D. Ferencek, Y. Gershtein, R. Gray, E. Halkiadakis, D. Hidas, E. Hughes, S. Kaplan, R. Kunnawalkam Elayavalli, A. Lath, K. Nash, S. Panwalkar, M. Park, S. Salur, S. Schnetzer, D. Sheffield, S. Somalwar, R. Stone, S. Thomas, P. Thomassen, M. Walker, M. Foerster, G. Riley, K. Rose, S. Spanier, A. York, O. Bouhali, A. Castaneda Hernandez, M. Dalchenko, M. De Mattia, A. Delgado, S. Dildick, R. Eusebi, J. Gilmore, T. Kamon, V. Krutelyov, R. Mueller, I. Osipenkov, Y. Pakhotin, R. Patel, A. Perloff, A. Rose, A. Safonov, A. Tatarinov, K. A. Ulmer, N. Akchurin, C. Cowden, J. Damgov, C. Dragoiu, P. R. Dudero, J. Faulkner, S. Kunori, K. Lamichhane, S. W. Lee, T. Libeiro, S. Undleeb, I. Volobouev, E. Appelt, A. G. Delannoy, S. Greene, A. Gurrola, R. Janjam, W. Johns, C. Maguire, Y. Mao, A. Melo, H. Ni, P. Sheldon, B. Snook, S. Tuo, J. Velkovska, Q. Xu, M. W. Arenton, B. Cox, B. Francis, J. Goodell, R. Hirosky, A. Ledovskoy, H. Li, C. Lin, C. Neu, T. Sinthuprasith, X. Sun, Y. Wang, E. Wolfe, J. Wood, F. Xia, C. Clarke, R. Harr, P. E. Karchin, C. Kottachchi Kankanamge Don, P. Lamichhane, J. Sturdy, D. A. Belknap, D. Carlsmith, M. Cepeda, S. Dasu, L. Dodd, S. Duric, E. Friis, B. Gomber, M. Grothe, R. Hall-Wilton, M. Herndon, A. Hervé, P. Klabbers, A. Lanaro, A. Levine, K. Long, R. Loveless, A. Mohapatra, I. Ojalvo, T. Perry, G. A. Pierro, G. Polese, T. Ruggles, T. Sarangi, A. Savin, A. Sharma, N. Smith, W. H. Smith, D. Taylor, N. Woods, [Authorinst]The CMS Collaboration

**Affiliations:** 1 Yerevan Physics Institute, Yerevan, Armenia; 2 Institut für Hochenergiephysik der OeAW, Wien, Austria; 3 National Centre for Particle and High Energy Physics, Minsk, Belarus; 4 Universiteit Antwerpen, Antwerpen, Belgium; 5 Vrije Universiteit Brussel, Brussel, Belgium; 6 Université Libre de Bruxelles, Bruxelles, Belgium; 7 Ghent University, Ghent, Belgium; 8 Université Catholique de Louvain, Louvain-la-Neuve, Belgium; 9 Université de Mons, Mons, Belgium; 10 Centro Brasileiro de Pesquisas Fisicas, Rio de Janeiro, Brazil; 11 Universidade do Estado do Rio de Janeiro, Rio de Janeiro, Brazil; 12 Universidade Estadual Paulista, Universidade Federal do ABC, São Paulo, Brazil; 13 Institute for Nuclear Research and Nuclear Energy, Sofia, Bulgaria; 14 University of Sofia, Sofia, Bulgaria; 15 Institute of High Energy Physics, Beijing, China; 16 State Key Laboratory of Nuclear Physics and Technology, Peking University, Beijing, China; 17 Universidad de Los Andes, Bogota, Colombia; 18 Faculty of Electrical Engineering, Mechanical Engineering and Naval Architecture, University of Split, Split, Croatia; 19 Faculty of Science, University of Split, Split, Croatia; 20 Institute Rudjer Boskovic, Zagreb, Croatia; 21 University of Cyprus, Nicosia, Cyprus; 22 Charles University, Prague, Czech Republic; 23 Academy of Scientific Research and Technology of the Arab Republic of Egypt, Egyptian Network of High Energy Physics, Cairo, Egypt; 24 National Institute of Chemical Physics and Biophysics, Tallinn, Estonia; 25 Department of Physics, University of Helsinki, Helsinki, Finland; 26 Helsinki Institute of Physics, Helsinki, Finland; 27 Lappeenranta University of Technology, Lappeenranta, Finland; 28 DSM/IRFU, CEA/Saclay, Gif-sur-Yvette, France; 29 Laboratoire Leprince-Ringuet, Ecole Polytechnique, IN2P3-CNRS, Palaiseau, France; 30 Institut Pluridisciplinaire Hubert Curien, Université de Strasbourg, Université de Haute Alsace Mulhouse, CNRS/IN2P3, Strasbourg, France; 31 Centre de Calcul de l’Institut National de Physique Nucleaire et de Physique des Particules, CNRS/IN2P3, Villeurbanne, France; 32 Institut de Physique Nucléaire de Lyon, Université de Lyon, Université Claude Bernard Lyon 1, CNRS-IN2P3, Villeurbanne, France; 33 Georgian Technical University, Tbilisi, Georgia; 34 Tbilisi State University, Tbilisi, Georgia; 35 I. Physikalisches Institut, RWTH Aachen University, Aachen, Germany; 36 III. Physikalisches Institut A, RWTH Aachen University, Aachen, Germany; 37 III. Physikalisches Institut B, RWTH Aachen University, Aachen, Germany; 38 Deutsches Elektronen-Synchrotron, Hamburg, Germany; 39 University of Hamburg, Hamburg, Germany; 40 Institut für Experimentelle Kernphysik, Karlsruhe, Germany; 41 Institute of Nuclear and Particle Physics (INPP), NCSR Demokritos, Aghia Paraskevi, Greece; 42 National and Kapodistrian University of Athens, Athens, Greece; 43 University of Ioánnina, Ioannina, Greece; 44 Wigner Research Centre for Physics, Budapest, Hungary; 45 Institute of Nuclear Research ATOMKI, Debrecen, Hungary; 46 University of Debrecen, Debrecen, Hungary; 47 National Institute of Science Education and Research, Bhubaneswar, India; 48 Panjab University, Chandigarh, India; 49 University of Delhi, Delhi, India; 50 Saha Institute of Nuclear Physics, Kolkata, India; 51 Bhabha Atomic Research Centre, Mumbai, India; 52 Tata Institute of Fundamental Research, Mumbai, India; 53 Indian Institute of Science Education and Research (IISER), Pune, India; 54 Institute for Research in Fundamental Sciences (IPM), Tehran, Iran; 55 University College Dublin, Dublin, Ireland; 56 INFN Sezione di Bari, Università di Bari, Politecnico di Bari, Bari, Italy; 57 INFN Sezione di Bologna, Università di Bologna, Bologna, Italy; 58 INFN Sezione di Catania, Università di Catania, Catania, Italy; 59 INFN Sezione di Firenze, Università di Firenze, Firenze, Italy; 60 INFN Laboratori Nazionali di Frascati, Frascati, Italy; 61 INFN Sezione di Genova, Università di Genova, Genova, Italy; 62 INFN Sezione di Milano-Bicocca, Università di Milano-Bicocca, Milan, Italy; 63 INFN Sezione di Napoli, Università di Napoli ‘Federico II’, Naples, Italy, Università della Basilicata, Potenza, Italy, Università G. Marconi, Rome, Italy; 64 INFN Sezione di Padova, Università di Padova, Padova, Italy, Università di Trento, Trento, Italy; 65 INFN Sezione di Pavia, Università di Pavia, Pavia, Italy; 66 INFN Sezione di Perugia, Università di Perugia, Perugia, Italy; 67 INFN Sezione di Pisa, Università di Pisa, Scuola Normale Superiore di Pisa, Pisa, Italy; 68 INFN Sezione di Roma, Università di Roma, Rome, Italy; 69 INFN Sezione di Torino, Università di Torino, Turin, Italy, Università del Piemonte Orientale, Novara, Italy; 70 INFN Sezione di Trieste, Università di Trieste, Trieste, Italy; 71 Kangwon National University, Chunchon, Korea; 72 Kyungpook National University, Taegu, Korea; 73 Chonbuk National University, Chonju, Korea; 74 Institute for Universe and Elementary Particles, Chonnam National University, Kwangju, Korea; 75 Korea University, Seoul, Korea; 76 Seoul National University, Seoul, Korea; 77 University of Seoul, Seoul, Korea; 78 Sungkyunkwan University, Suwon, Korea; 79 Vilnius University, Vilnius, Lithuania; 80 National Centre for Particle Physics, Universiti Malaya, Kuala Lumpur, Malaysia; 81 Centro de Investigacion y de Estudios Avanzados del IPN, Mexico City, Mexico; 82 Universidad Iberoamericana, Mexico City, Mexico; 83 Benemerita Universidad Autonoma de Puebla, Puebla, Mexico; 84 Universidad Autónoma de San Luis Potosí, San Luis Potosí, Mexico; 85 University of Auckland, Auckland, New Zealand; 86 University of Canterbury, Christchurch, New Zealand; 87 National Centre for Physics, Quaid-I-Azam University, Islamabad, Pakistan; 88 National Centre for Nuclear Research, Swierk, Poland; 89 Institute of Experimental Physics, Faculty of Physics, University of Warsaw, Warsaw, Poland; 90 Laboratório de Instrumentação e Física Experimental de Partículas, Lisbon, Portugal; 91 Joint Institute for Nuclear Research, Dubna, Russia; 92 Petersburg Nuclear Physics Institute, Gatchina, St. Petersburg, Russia; 93 Institute for Nuclear Research, Moscow, Russia; 94 Institute for Theoretical and Experimental Physics, Moscow, Russia; 95 National Research Nuclear University ‘Moscow Engineering Physics Institute’ (MEPhI), Moscow, Russia; 96 P. N. Lebedev Physical Institute, Moscow, Russia; 97 Skobeltsyn Institute of Nuclear Physics, Lomonosov Moscow State University, Moscow, Russia; 98 State Research Center of Russian Federation, Institute for High Energy Physics, Protvino, Russia; 99 Faculty of Physics and Vinca Institute of Nuclear Sciences, University of Belgrade, Belgrade, Serbia; 100 Centro de Investigaciones Energéticas Medioambientales y Tecnológicas (CIEMAT), Madrid, Spain; 101 Universidad Autónoma de Madrid, Madrid, Spain; 102 Universidad de Oviedo, Oviedo, Spain; 103 Instituto de Física de Cantabria (IFCA), CSIC-Universidad de Cantabria, Santander, Spain; 104 CERN, European Organization for Nuclear Research, Geneva, Switzerland; 105 Paul Scherrer Institut, Villigen, Switzerland; 106 Institute for Particle Physics, ETH Zurich, Zurich, Switzerland; 107 Universität Zürich, Zurich, Switzerland; 108 National Central University, Chung-Li, Taiwan; 109 National Taiwan University (NTU), Taipei, Taiwan; 110 Department of Physics, Faculty of Science, Chulalongkorn University, Bangkok, Thailand; 111 Cukurova University, Adana, Turkey; 112 Physics Department, Middle East Technical University, Ankara, Turkey; 113 Bogazici University, Istanbul, Turkey; 114 Istanbul Technical University, Istanbul, Turkey; 115 Institute for Scintillation Materials of National Academy of Science of Ukraine, Kharkov, Ukraine; 116 National Scientific Center, Kharkov Institute of Physics and Technology, Kharkov, Ukraine; 117 University of Bristol, Bristol, UK; 118 Rutherford Appleton Laboratory, Didcot, UK; 119 Imperial College, London, UK; 120 Brunel University, Uxbridge, UK; 121 Baylor University, Waco, USA; 122 The University of Alabama, Tuscaloosa, USA; 123 Boston University, Boston, USA; 124 Brown University, Providence, USA; 125 University of California, Davis, Davis, USA; 126 University of California, Los Angeles, USA; 127 University of California, Riverside, Riverside, USA; 128 University of California, San Diego, La Jolla, USA; 129 University of California, Santa Barbara, Santa Barbara, USA; 130 California Institute of Technology, Pasadena, USA; 131 Carnegie Mellon University, Pittsburgh, USA; 132 University of Colorado Boulder, Boulder, USA; 133 Cornell University, Ithaca, USA; 134 Fermi National Accelerator Laboratory, Batavia, USA; 135 University of Florida, Gainesville, USA; 136 Florida International University, Miami, USA; 137 Florida State University, Tallahassee, USA; 138 Florida Institute of Technology, Melbourne, USA; 139 University of Illinois at Chicago (UIC), Chicago, USA; 140 The University of Iowa, Iowa City, USA; 141 Johns Hopkins University, Baltimore, USA; 142 The University of Kansas, Lawrence, USA; 143 Kansas State University, Manhattan, USA; 144 Lawrence Livermore National Laboratory, Livermore, USA; 145 University of Maryland, College Park, USA; 146 Massachusetts Institute of Technology, Cambridge, USA; 147 University of Minnesota, Minneapolis, USA; 148 University of Mississippi, Oxford, USA; 149 University of Nebraska-Lincoln, Lincoln, USA; 150 State University of New York at Buffalo, Buffalo, USA; 151 Northeastern University, Boston, USA; 152 Northwestern University, Evanston, USA; 153 University of Notre Dame, Notre Dame, USA; 154 The Ohio State University, Columbus, USA; 155 Princeton University, Princeton, USA; 156 University of Puerto Rico, Mayaguez, USA; 157 Purdue University, West Lafayette, USA; 158 Purdue University Calumet, Hammond, USA; 159 Rice University, Houston, USA; 160 University of Rochester, Rochester, USA; 161 Rutgers, The State University of New Jersey, Piscataway, USA; 162 University of Tennessee, Knoxville, USA; 163 Texas A&M University, College Station, USA; 164 Texas Tech University, Lubbock, USA; 165 Vanderbilt University, Nashville, USA; 166 University of Virginia, Charlottesville, USA; 167 Wayne State University, Detroit, USA; 168 University of Wisconsin-Madison, Madison, WI USA; 169 CERN, Geneva, Switzerland

## Abstract

A measurement of the forward–backward asymmetry ${A}_{\mathrm{FB}}$ of oppositely charged lepton pairs ($\mu \mu$ and $\mathrm{e}\mathrm{e}$) produced via $\mathrm{Z}/\gamma ^*$ boson exchange in pp collisions at $\sqrt{s} = 8$ $\,\mathrm{TeV}$ is presented. The data sample corresponds to an integrated luminosity of 19.7$\,\mathrm{fb}^{-1}$ collected with the CMS detector at the LHC. The measurement of ${A}_{\mathrm{FB}}$ is performed for dilepton masses between 40$\,\text {GeV}$ and 2$\,\mathrm{TeV}$ and for dilepton rapidity up to 5. The ${A}_{\mathrm{FB}}$ measurements as a function of dilepton mass and rapidity are compared with the standard model predictions.

## Introduction

A forward–backward asymmetry ${A}_{\mathrm{FB}}$ in the production of Drell–Yan lepton pairs arises from the presence of both vector and axial-vector couplings of electroweak bosons to fermions. For a given dilepton invariant mass *M* the differential cross section at the parton level at leading order (LO) can be expressed as

1 $$\begin{aligned} \frac{\mathrm{d}\sigma }{\mathrm{d}(\cos \theta ^{*})} = A (1 + \cos ^{2} \theta ^{*}) + B \cos \theta ^{*}, \end{aligned}$$

where $\theta ^{*}$ represents the emission angle of the negatively charged lepton relative to the quark momentum in the rest frame of the dilepton system, and *A* and *B* are parameters that depend on *M*, the electroweak mixing angle $\theta _\mathrm{W}$, and the weak isospin and charge of the incoming and outgoing fermions. The ${A}_{\mathrm{FB}}$ quantity is

2 $$\begin{aligned} {A}_\mathrm {FB} = \frac{\sigma _\mathrm {F}-\sigma _\mathrm {B}}{\sigma _\mathrm {F}+\sigma _\mathrm {B}}, \end{aligned}$$

where $\sigma _\mathrm {F}$ ($\sigma _\mathrm {B}$) is the total cross section for the forward (backward) events, defined by $\cos \theta ^{*} > 0$ ($\cos \theta ^{*} < 0$). ${A}_{\mathrm{FB}}$ depends on *M* , quark flavor, and the electroweak mixing angle $\theta _W$. Near the Z boson mass peak ${A}_{\mathrm{FB}}$ is close to zero because of the small value of the lepton vector coupling to Z bosons. Due to weak-electromagnetic interference, ${A}_{\mathrm{FB}}$ is large and negative for *M* below the Z peak ($M < 80$ $\,\text {GeV}$) and large and positive above the $\mathrm{Z}$ peak ($M > 110$ $\,\text {GeV}$). Deviations from the SM predictions could result from the presence of additional neutral gauge bosons [1–5], quark-lepton compositeness [6], supersymmetric particles, or extra dimensions [7]. Around the $\mathrm{Z}$ peak, measurements of ${A}_{\mathrm{FB}}$ can also be used to extract the effective weak mixing angle $\sin ^2\theta ^\text {eff}_{\text {lept}}(m_{\mathrm{Z}})$ [8, 9] as well as the $\mathrm{u}$ and $\mathrm{d}$ quark weak coupling [9–12].

To reduce the uncertainties due to the transverse momentum (${p}_{\mathrm{T}}$) of the incoming quarks, this measurement uses the Collins–Soper (CS) frame [13]. In this frame, $\theta ^{*}_{\mathrm {CS}}$ is defined as the angle between the negatively charged lepton momentum and the axis that bisects the angle between the quark momentum direction and the opposite direction to the antiquark momentum. In the laboratory frame, $\theta ^{*}_{\mathrm {CS}}$ is calculated as

3 $$\begin{aligned} \cos \theta ^{*}_{\mathrm {CS}}=\frac{2(P^+_{1}P^-_{2}-P^-_{1}P^+_{2})}{\sqrt{Q^2(Q^2+Q_\mathrm {T}^2)}}, \end{aligned}$$

where *Q* and $Q_\mathrm {T}$ represent the four-momentum and the ${p}_{\mathrm{T}}$ of the dilepton system, respectively, while $P_{1}$ ($P_{2}$) represents the four-momentum of $\ell ^{-}$ ($\ell ^{+}$) with $P^\pm _{i} = (E_{i}\pm P_{z,i})/\sqrt{2}$, and $E_{i}$ represents the energy of the lepton.

The production of lepton pairs arises mainly from the annihilation of valence quarks with sea antiquarks. At the LHC, the quark and antiquark directions are not known for each collision because both beams consist of protons. In general, however, the quark carries more momentum than the antiquark as the antiquark must originate from the parton sea. Therefore, on average, the dilepton system is boosted in the direction of the valence quark [2, 14, 15]. In this paper, the positive axis is defined to be along the boost direction using the following transformation on an event-by-event basis:

4 $$\begin{aligned} \cos \theta ^{*}_{\mathrm {CS}}\rightarrow \frac{|Q_{ z} |}{Q_{z}}\cos \theta ^{*}_{\mathrm {CS}}, \end{aligned}$$

where $Q_z$ is the longitudinal momentum of the dilepton system. The fraction of events for which the quark direction is the same as the direction of the boost depends on *M* and increases with the absolute value of the dilepton rapidity $y = \frac{1}{2}\ln [(E+Q_z)/(E-Q_z)]$.

${A}_{\mathrm{FB}}$ was previously measured by the CMS [16] and ATLAS [8] experiments using data samples collected at $\sqrt{s} = 7$ $\,\mathrm{TeV}$. The techniques used in this analysis are similar to those used in the previous CMS measurement at 7$\,\mathrm{TeV}$, and the rapidity range of this measurement is extended to $|y |=5$ by including electrons in the forward calorimeter. Since large Z boson rapidities are better correlated with the direction of the valence quark, ${A}_{\mathrm{FB}}$ is measured as a function of the invariant mass and the rapidity of Z boson. The number of selected events at 8$\,\mathrm{TeV}$ is about a factor of 5 larger than the number of events at 7$\,\mathrm{TeV}$. The larger data sample collected at 8$\,\mathrm{TeV}$ extends the measurement of ${A}_{\mathrm{FB}}$ in the high-mass region where the number of events in the 7$\,\mathrm{TeV}$ samples was limited.

## The CMS detector

The central feature of the CMS detector is a superconducting solenoid with a 6 m internal diameter that provides a magnetic field of 3.8 T. Inside the solenoid are a silicon pixel and strip tracker, a lead tungstate crystal electromagnetic calorimeter (ECAL), and a brass and scintillator hadron calorimeter, each composed of a barrel and two endcap sections. Extensive forward calorimetry complements the coverage provided by the barrel and endcap calorimeters. Outside the solenoid, gas-ionization detectors embedded in the steel flux-return yoke are used to measure muons.

Muons are measured in the pseudorapidity [17] range $|\eta |<2.4$ using the silicon tracker and muon systems. The muon detectors are constructed using three different technologies: drift tubes for $|\eta |<1.2$, cathode strip chambers for $0.9<|\eta |<2.4$, and resistive plate chambers for $|\eta |< 1.6$. Matching muons to tracks measured in the silicon tracker results in a relative ${p}_{\mathrm{T}}$ resolution of 1.3–2.0 % in the barrel, and better than 6 % in the endcaps for muons with $20< {p}_{\mathrm{T}} < 100$ $\,\text {GeV}$  [18].

Electrons are measured in the range $|\eta |<2.5$ using both the tracking system and the ECAL. The energy resolution for electrons produced in Z boson decays varies from 1.7 % in the barrel ($|\eta |<1.48$) to 4.5 % in the endcap region ($|\eta |>1.48$) [19].

The $\eta$ coverage of the CMS detector is extended up to $|\eta |=5$ by the hadron forward (HF) calorimeters [20]. The HF is constructed from steel absorbers as shower initiators and quartz fibers as active material. Half of the fibers extend over the full depth of the detector (long fibers) while the other half does not cover the first 22 cm measured from the front face (short fibers). As the two sets of fibers are read out separately, electromagnetic showers can be distinguished from hadronic showers. Electrons in the HF are measured in the range $3< |\eta | < 5$. The energy resolution for HF electrons is ${\sim } 32\%$ at 50 $\,\text {GeV}$ and the angular resolution is up to 0.05 in $\eta$ and $\phi$.

The CMS experiment uses a two-level trigger system. The level-1 trigger, composed of custom-designed processing hardware, selects events of interest based on information from the muon detectors and calorimeters [21]. The high-level trigger is software based, running a faster version of the offline reconstruction code on the full detector information, including the tracker [22]. A more detailed description of the CMS detector, together with a definition of the coordinate system used and the relevant kinematic variables, can be found in Ref. [17].

## Data and Monte Carlo samples

The analysis is performed using the pp collision data collected with the CMS detector in 2012 at a center-of-mass energy of 8$\,\mathrm{TeV}$. The total integrated luminosity for the entire data set amounts to 19.7$\,\mathrm{fb}^{-1}$.

The simulated $\mathrm{Z}/\gamma ^* \rightarrow \mu \mu$ and $\mathrm{Z}/\gamma ^* \rightarrow \mathrm{e}\mathrm{e}$ signal samples are generated at next-to-leading order (NLO) based in perturbative QCD using powheg  [23–26] with the NLO CT10 parton distribution functions (PDFs) [27]. The parton showering and hadronization are simulated using the pythia v6.426 [28] generator with the Z2* tune [29].

The background processes, $\mathrm{Z}/\gamma ^* \rightarrow \tau \tau$, $\mathrm{t}\overline{\mathrm{t}}$, $\mathrm{t} {\mathrm{W}^{-}}$ and $\overline{\mathrm{t}} {\mathrm{W}^{+}}$, are generated with powheg, and the inclusive W production with MadGraph  [30]. The backgrounds from WW, WZ, and ZZ production are generated using pythia v6.426. The $\tau$ lepton decays in the background processes are simulated using tauola  [31]. For all processes, the detector response is simulated using a detailed description of the CMS detector based on the Geant4 package [32, 33]. GFlash [34] is used for the HF [35], and the event reconstruction is performed with the same algorithms used for the data. The data contain multiple proton-proton interactions per bunch crossing (pileup) with an average value of 21. A pileup reweighting procedure is applied to the Monte Carlo (MC) simulation so the pileup distribution matches the data.

## Event selection

The inclusive dimuon events are selected by a trigger that requires two muons, the leading one with ${p}_{\mathrm{T}} >17$ $\,\text {GeV}$ and the second one with ${p}_{\mathrm{T}} >8$ $\,\text {GeV}$. Muons are selected offline by the standard CMS muon identification [18], which requires at least one muon chamber hit in the global muon track fit, muon segments in at least two muon stations, at least one hit in the pixel detector, more than five inner tracker layers with hits, and a $\chi ^{2}/\mathrm {dof}$ less than 10 for the global muon fit. The vertex with the highest ${p}_{\mathrm{T}}$ sum for associated tracks is defined as the primary vertex. The distance between the muon candidate trajectories and the primary vertex is required to be smaller than 2 mm in the transverse plane and smaller than 5 mm in the longitudinal direction. This requirement significantly reduces the background from cosmic ray muons. To remove muons produced during jet fragmentation, the fractional track isolation, $\sum {p}_{\mathrm{T}} ^{\text {trk}} / {p}_{\mathrm{T}} ^{\mu }$, is required to be smaller than 0.1, where the sum runs over all tracks originating from the primary vertex within a cone of $\Delta R= \sqrt{{(\Delta \eta )^2 + (\Delta \phi )^2}}<0.3$ around each of the identified muons. Furthermore, each selected muon is required to have ${p}_{\mathrm{T}} >20$ $\,\text {GeV}$ and $|\eta |<2.4$.

The inclusive dielectron events include electrons that are produced in an extended lepton pseudorapidity range, $|\eta |<5$. The events with dilepton rapidity $|y| < 2.4$ are selected by triggers requiring either two central electrons, $|\eta |<2.4$, with ${p}_{\mathrm{T}} >17$ and $>8$ $\,\text {GeV}$. In the analysis, the central electron candidates are required to have ${p}_{\mathrm{T}} >20$ $\,\text {GeV}$, have opposite charges, and to pass tight electron identification and isolation requirements [19]. The particle-flow (PF) event reconstruction [36, 37] consists of reconstructing and identifying each single particle with an optimized combination of all subdetector information. In this process, the identification of the particle type (photon, electron, muon, charged hadron, or neutral hadron) plays an important role in the determination of the particle direction and energy. The fractional PF isolation, $\sum {p}_{\mathrm{T}} ^{\mathrm {PF}} / {p}_{\mathrm{T}} ^{\mathrm{e}}$, is required to be smaller than 0.1. The isolation variable is calculated from the energy sum over all PF candidates within a cone of size 0.3 around each of the identified electrons. This sample is used to perform the analysis for the dilepton rapidity, $|y |<2.4$.

For the events with dilepton rapidity $2.4< |y| < 5$, one central ($|\eta |<2.4$) and one forward electron ($3<|\eta |<5$) are used requiring one isolated central electron trigger with ${p}_{\mathrm{T}} >27$ $\,\text {GeV}$. In this case, the central (forward) electron candidate is required to have ${p}_{\mathrm{T}} > 30\,(20)$ $\,\text {GeV}$, as well as to pass stringent electron identification and isolation requirements (forward electron identification criteria). Since the $2.4<|\eta |<3$ region is outside the tracker acceptance, the particle flow variables cannot be defined in this region, and are therefore not considered in the analysis.

Forward electron identification requires an isolated energy deposition in the core of the electron cluster [35]. To reduce the contribution from jet background in the forward region, both electrons are required to be on the same side of the detector ($\eta _{\mathrm{e}_{1}} \, \eta _{\mathrm{e}_{2}} >0$) and almost back-to-back in azimuth ($|\Delta \phi (\mathrm{e}_{1},\mathrm{e}_{2}) |> 2\pi /3$). Because the forward electrons do not have charge information, no oppositely-charged requirement is applied.

After the event selection, about 8 million $\mu \mu$ and 4.3 million $\mathrm{e}\mathrm{e}$ events remain with $|y |<2.4$, and 0.5 million $\mathrm{e}\mathrm{e}$ events with $2.4<|y |<5$.

## Simulation corrections

Scale factors are derived and applied to the simulated MC events to account for differences of detector performance between data and the MC simulation. The efficiencies for the trigger, lepton identification, and lepton isolation are measured using a “tag-and-probe” method [18, 38] for both data and simulation. For the muon channel, the trigger efficiency is measured as a function of $\eta$ only because the ${p}_{\mathrm{T}}$ dependence is small for ${p}_{\mathrm{T}} >20$ $\,\text {GeV}$, while in the electron channel the efficiency is measured as a function of $E_{\mathrm T}$ and $\eta$. Similarly, the identification and isolation efficiencies for the muons and central electrons are measured in data and simulation as a function of ${p}_{\mathrm{T}}$ and $\eta$. The difference in trigger efficiency between data and simulation is 1 to 4 % for the muon channel, depending on the $\eta$ region, and less than 1 % for the electron channel. The differences in the muon identification and isolation efficiencies are less than 1 %. For central electrons the absolute difference is at the 5 % level in the barrel and increases to 12 % in the endcaps.

For forward electrons, the identification efficiency is measured as a function of ${E}_{\mathrm{T}}$ and $\eta$. We observe a 9 to 18 % difference in the identification efficiency between data and MC simulation. The simulation is scaled using these factors to reproduce the data. Forward electrons require additional corrections in GFlash simulation in order to match the $\eta$ distribution of the data. Furthermore, a global normalization factor of $0.6 \pm 0.3$ is applied to account for the data/simulation difference in the event yields in HF. Its effect is negligible in the ${A}_{\mathrm{FB}}(M)$ measurement.

The muon momentum and electron energy scales are affected by detector misalignment and imperfect calibration, which cause a degradation in the energy measurements and the measurement of ${A}_{\mathrm{FB}}$. Such effects are accounted for by additional momentum and energy corrections, which are applied to muons and electrons in both data and simulation. It has been shown [18] that the primary cause of the bias in the reconstructed muon momentum is the misalignment of the tracking system. To remove this bias, a muon momentum correction extracted as a function of the muon charge, $\theta$, and $\phi$ [39] is applied for both data and MC events. The overall muon momentum corrections for muons with ${p}_{\mathrm{T}} >20$ $\,\text {GeV}$ are measured with a precision of better than 0.04 %.

For central electrons, an ECAL energy scale correction is applied. The overall energy scale for electrons with $7< {p}_{\mathrm{T}} <70$ $\,\text {GeV}$ is measured with a precision better than 0.3 % [19]. To match the electron energy resolutions in data, additional smearing is applied to the energy of central electrons in the MC simulation. For forward electrons, the predicted energy of the forward electron is calculated using Z boson mass, the energy of the central electron, and the angular positions ($\eta$ and $\phi$) of central and forward electrons. The residual energy correction for forward electrons as a function of ${E}_{\mathrm{T}}$ is determined from the average of the difference between the reconstructed energy and the predicted energy. The corrections are applied in data and simulation as a function of the electron ${E}_{\mathrm{T}}$ and range between $-18$ and $+12\,\%$. The energy resolution of the forward electron in the MC simulation is also tuned to match the data.

**Fig. 1:**
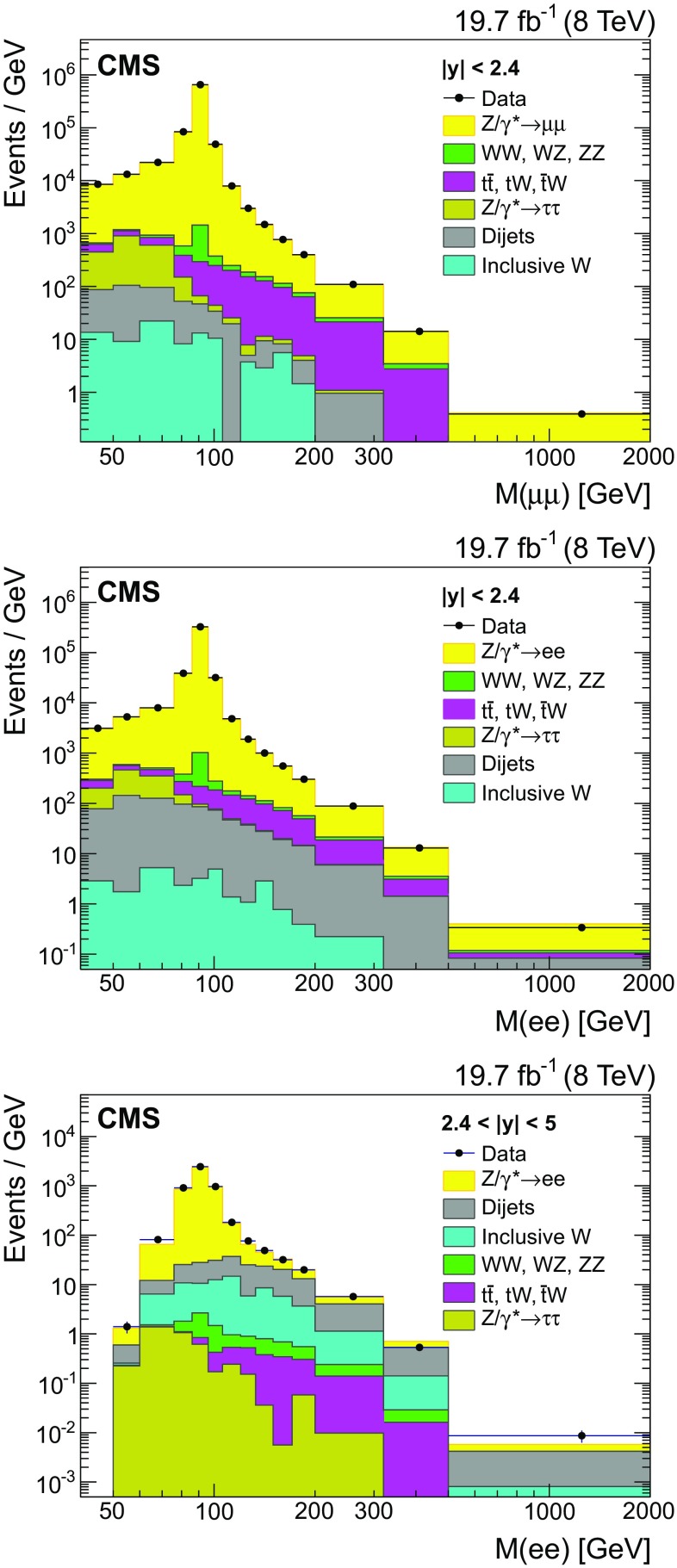
The invariant mass distributions for $\mu \mu$ (*top*), $\mathrm{e}\mathrm{e}$ (*middle*) events with $|y |<2.4$, and $\mathrm{e}\mathrm{e}$ (*bottom*) events with $2.4<|y |<5$. Only statistical uncertainties are shown. The stacked histograms represent the sum of the background contributions and the signal

**Fig. 2:**
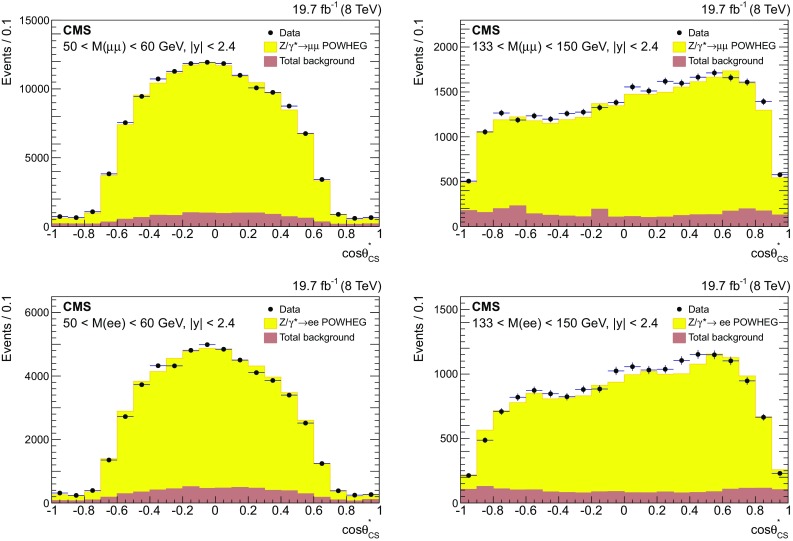
The $\cos \theta ^{*}_\mathrm {CS}$ distributions for $\mu \mu$ ($\mathrm{e}\mathrm{e}$) events are presented in the *top* (*bottom*) *panels*. Only statistical uncertainties are shown. The stacked histograms represent the sum of the background contribution and the signal. The plots on the *left* (*right*) *panels* correspond to events with dilepton invariant mass $50< M < 60$ $\,\text {GeV}$ ($133< M < 150$ $\,\text {GeV}$)

## Backgrounds

The main sources of background at low dilepton mass are $\mathrm{Z}/\gamma ^{*} \rightarrow \tau \tau$ events and QCD dijet events. At high mass, the main background comes from t$\bar{\text {t}}$ events. The diboson (WW, WZ, ZZ) and inclusive W background contributions are small. The background contributions are estimated versus *M* and $|y |$ for forward and backward events separately. Different techniques are used for estimating background contributions in the muon and electron channels.

The dijet background for both muon and electron channels is estimated with data using control samples. The muon channel uses same-sign dimuon events, which mostly originate from dijets. The number of same-sign events after the final event selection is used to estimate the number of opposite-sign dimuons that originate from dijets. The contribution from the diboson process is subtracted in the same-sign events using MC simulation.

For the electron channel, a fitting method is used to estimate the dijet background. The kinematic distributions of the $\mathrm{e}\mathrm{e}$ events in *M* and $|y |$ are fitted with a sum of signal and background templates to determine the dijet component. A signal template is extracted from the $\mathrm{Z}/\gamma ^{*} \rightarrow \mathrm{e}\mathrm{e}$ MC sample. A background template is obtained by applying a reverse isolation requirement on the central electron in data. The signal and non-QCD background contributions, which are small, are subtracted from this nonisolated electron sample using simulation.

In the muon channel, events selected with an $\mathrm{e}\mu$ lepton pair are used to determine the backgrounds from $\mathrm{Z}/\gamma ^{*} \rightarrow \tau \tau$, $\mathrm{t}\overline{\mathrm{t}}$, W+jets, $\mathrm{t} \mathrm{W}$, and $\overline{\mathrm{t}} \mathrm{W}$ processes. The overall rate for $\mu \mu$ background events from these sources is proportional to the number of observed $\mathrm{e}\mu$ events. Here the MC simulation is used only to calculate the ratio of $\mu \mu$ events to $\mathrm{e}\mu$ events. The background rate extracted with this method is in agreement with MC simulations. Therefore, in the electron analysis these backgrounds are modelled using MC simulations. The cross sections are normalized to next-to-next-to-leading-order fewz predictions [40]. Also, the diboson backgrounds are estimated using MC simulation for both the muon and electron channels.

The invariant mass distributions for $\mu \mu$ and $\mathrm{e}\mathrm{e}$ events in two $|y |$ ranges are shown in Fig. 1, which also includes the MC predictions for both the signal and estimated background contributions. The MC predictions are normalized using the cross section for each process and the integrated luminosity.

## Measurement of ${A}_{\mathrm{FB}}$

The events are assigned to “forward” or “backward” regions as described in Sect. 1. ${A}_{\mathrm{FB}}$ is measured using the selected dilepton events as a function of dilepton mass in five regions of absolute rapidity: 0–1, 1–1.25, 1.25–1.5, 1.5–2.4, and 2.4–5. The most forward region has 7 mass bins, from 40 to 320$\,\text {GeV}$, while the others have 14 mass bins, which extend up to 2$\,\mathrm{TeV}$. The shape of the $\cos \theta ^{*}_\mathrm {CS}$ distribution changes with the dilepton mass. The top panels of Fig. 2 show the reconstructed $\cos \theta ^{*}_{\mathrm {CS}}$ distributions for $\mu \mu$ events, with $|y | < 2.4$. The bottom panels show the reconstructed $\cos \theta ^{*}_\mathrm {CS}$ for $\mathrm{e}\mathrm{e}$ events, with $|y | < 2.4$. The distributions are shown for two representative mass bins. The distributions for dilepton events at low mass ($50< M < 60$ $\,\text {GeV}$) are shown in the left panels, and at high mass ($133< M < 150$ $\,\text {GeV}$) in the right panels. The MC predictions are normalized to the integrated luminosity of the data.

The measured ${A}_{\mathrm{FB}}$ value is corrected for detector resolution, acceptance, efficiency, and the effect of final-state QED radiation (FSR) using a two-dimensional iterative unfolding method based on Bayes’ theorem [41, 42]. The ${A}_{\mathrm{FB}}$ quantity is unfolded to account for event migration between mass bins and between positive and negative $\cos \theta ^{*}_\mathrm {CS}$ region. Since the ambiguity of the quark direction is more significant at low $|y |$, the dilution of ${A}_{\mathrm{FB}}$ is larger in the low $|y |$ region.

**Table 1:** The maximum value of the systematic uncertainty in ${A}_{\mathrm{FB}}$ as a function of *M* from each source for different regions of $|y |$

Muon channel					
Systematic uncertainty	$|y |$ bins				
	0–1	1–1.25	1.25–1.5	1.5–2.4	
Background	0.062	0.080	0.209	0.051	
Momentum correction	0.006	0.015	0.020	0.022	
Unfolding	0.001	0.003	0.004	0.003	
Pileup reweighting	0.002	0.004	0.003	0.004	
Efficiency scale factors	<0.001	0.002	0.003	0.005	
PDFs	0.001	0.004	0.008	0.047	
FSR	<0.001	0.001	0.001	0.002	
Electron channel					
Systematic uncertainty	$|y |$ bins				
	0–1	1–1.25	1.25–1.5	1.5–2.4	2.4–5
Background	0.064	0.015	0.008	0.004	0.033
Energy correction	0.011	0.015	0.012	0.012	0.123
Unfolding	0.005	0.007	0.006	0.004	0.001
Pileup reweighting	0.003	0.002	0.002	0.001	0.007
Efficiency scale factors	<0.001	<0.001	<0.001	<0.001	0.008
Forward $\eta$ scale factor	–	–	–	–	0.002
Forward $\eta$ asymmetry	–	–	–	–	0.029
Global normalization factor	–	–	–	–	0.060
PDFs	0.002	0.004	0.005	0.008	0.014
FSR	<0.001	0.001	0.001	0.001	0.002

**Fig. 3:**
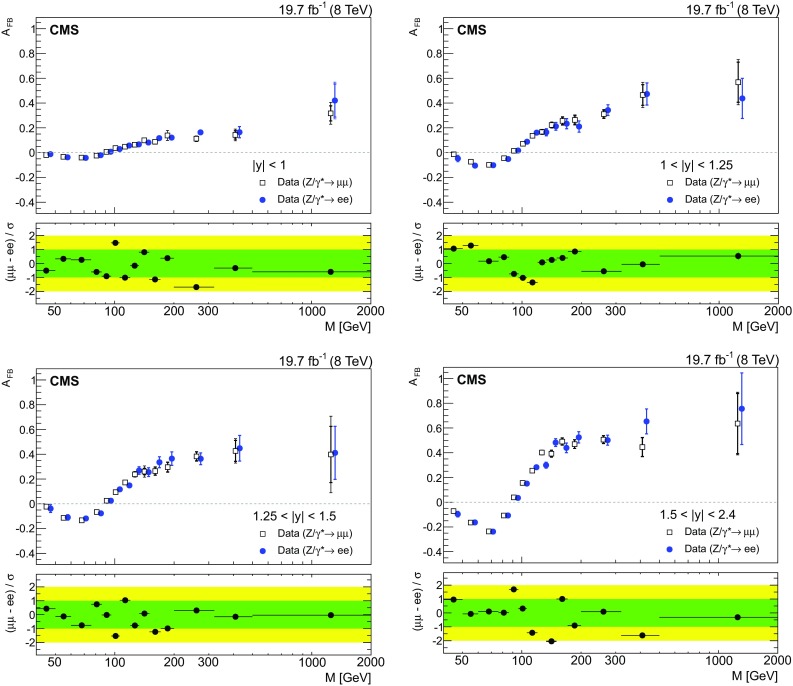
The unfolded ${A}_{\mathrm{FB}}$ distributions for muons (*open squares*) and electrons (*solid circles*) for the four central rapidity regions. The statistical (*thick vertical bar*) and statistical plus systematics (*thin vertical bar*) uncertainties are presented. The *solid circles* are shifted slightly to compare the result better. The *lower panel* in each plot shows the difference of the unfolded ${A}_{\mathrm{FB}}$ in muons and electrons divided by the total uncertainty (stat. $\oplus$ syst.)

**Fig. 4:**
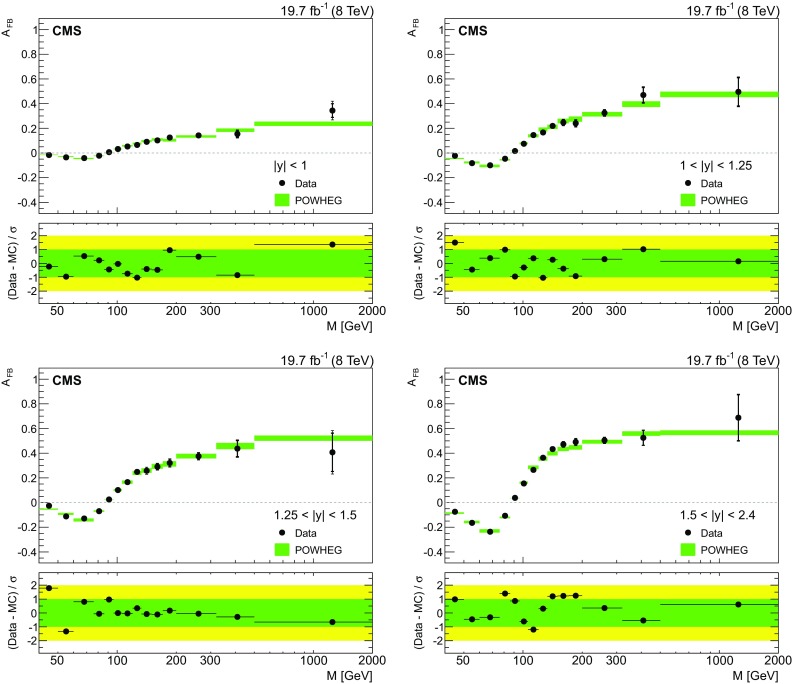
The combined (${{\mu }^{+}}{{\mu }^{-}}$ and $\mathrm{e}^{+}\mathrm{e}^{-}$ ) unfolded ${A}_{\mathrm{FB}}$ distributions in the four central rapidity regions. The statistical (*thick vertical bar*) and statistical plus systematics (*thin vertical bar*) uncertainties are presented. The measurements are compared with the prediction of powheg. The total uncertainties (considering the statistical, PDF, and scale uncertainties) in the powheg prediction are shown as *shaded* bands. The *lower panel* in each plot shows the difference of ${A}_{\mathrm{FB}}$ in data and prediction divided by the total uncertainty of data and prediction

**Fig. 5:**
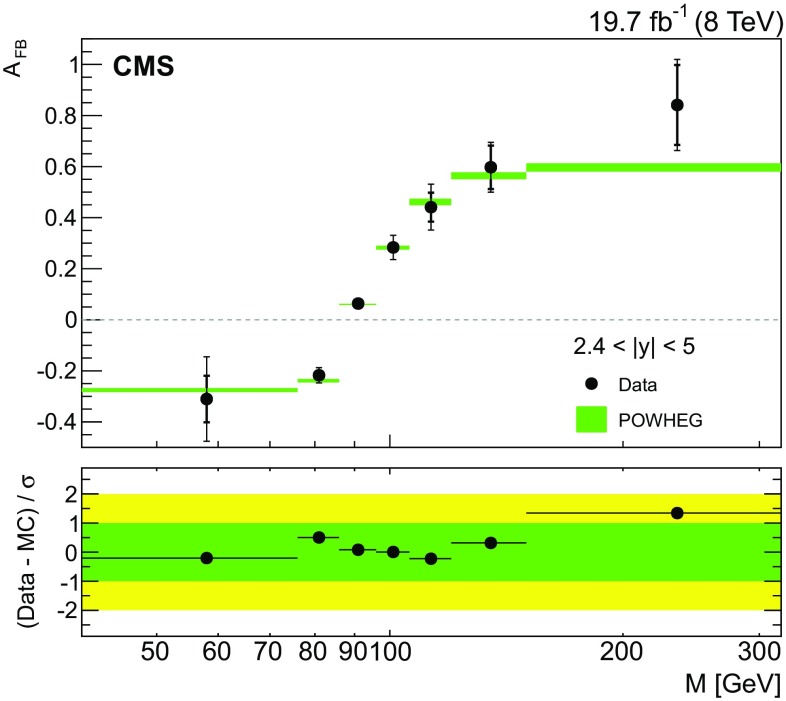
The unfolded ${A}_{\mathrm{FB}}$ distribution for the forward rapidity region ($2.4<|y |<5$) using one central electron ($|\eta |<2.4$) and one HF electron ($3<|\eta |<5$). The *inner thick vertical bars* correspond to the statistical uncertainty and the *outer thin vertical bars* to the total uncertainties. The measurements are compared with the prediction of powheg. The total uncertainties (considering the statistical, PDF, and scale uncertainties) in the powheg prediction are shown as *shaded* bands. The *lower panel* shows the difference of ${A}_{\mathrm{FB}}$ in data and prediction divided by the total uncertainty of data and prediction

**Table 2:** The combined ($\mathrm{e}\mathrm{e}$ and $\mu \mu$) ${A}_{\mathrm{FB}}$ measurements, with statistical and systematic uncertainties for the four rapidity regions with $|y |<2.4$. The ${A}_{\mathrm{FB}}$ quantity for $\mathrm{e}\mathrm{e}$ events is also shown for $2.4<|y |<5$

*M* ($\mathrm{GeV}$ )	${A}_{\mathrm{FB}}$ (data)	Stat. err	Syst. err	Tot. err	*M* ($\mathrm{GeV}$ )	${A}_{\mathrm{FB}}$ (data)	Stat. err	Syst. err	Tot. err
$|y |<1$					$1<|y |<1.25$				
40–50	$-$0.0167	0.0049	0.0045	0.0067	40–50	$-$0.0225	0.0108	0.0092	0.0142
50–60	$-$0.0355	0.0042	0.0031	0.0052	50–60	$-$0.0825	0.0092	0.0060	0.0110
60–76	$-$0.0415	0.0033	0.0031	0.0045	60–76	$-$0.0999	0.0071	0.0044	0.0084
76–86	$-$0.0221	0.0022	0.0019	0.0029	76–86	$-$0.0468	0.0048	0.0042	0.0064
86–96	0.0065	0.0004	0.0003	0.0005	86–96	0.0157	0.0009	0.0005	0.0011
96–106	0.0320	0.0020	0.0016	0.0025	96–106	0.0747	0.0046	0.0042	0.0063
106–120	0.0524	0.0037	0.0024	0.0045	106–120	0.1448	0.0085	0.0029	0.0089
120–133	0.0652	0.0065	0.0035	0.0074	120–133	0.1663	0.0152	0.0083	0.0174
133–150	0.0905	0.0081	0.0070	0.0108	133–150	0.2191	0.0185	0.0064	0.0195
150–171	0.1020	0.0104	0.0075	0.0128	150–171	0.2469	0.0243	0.0123	0.0272
171–200	0.1251	0.0129	0.0145	0.0194	171–200	0.2401	0.0272	0.0143	0.0308
200–320	0.1423	0.0112	0.0099	0.0149	200–320	0.3245	0.0257	0.0115	0.0282
320–500	0.1541	0.0268	0.0195	0.0331	320–500	0.4697	0.0609	0.0302	0.0680
500–2000	0.3437	0.0554	0.0514	0.0756	500–2000	0.4954	0.1145	0.0400	0.1213
$1.25<|y |<1.5$					$1.5<|y |<2.4$				
40–50	$-$0.0261	0.0114	0.0087	0.0144	40–50	$-$0.0747	0.0073	0.0049	0.0088
50–60	$-$0.1122	0.0098	0.0078	0.0125	50–60	$-$0.1645	0.0070	0.0053	0.0088
60–76	$-$0.1293	0.0077	0.0039	0.0086	60–76	$-$0.2365	0.0059	0.0052	0.0079
76–86	$-$0.0700	0.0052	0.0040	0.0065	76–86	$-$0.1071	0.0041	0.0057	0.0070
86–96	0.0249	0.0010	0.0007	0.0013	86–96	0.0379	0.0008	0.0009	0.0013
96–106	0.1012	0.0051	0.0044	0.0067	96–106	0.1546	0.0041	0.0057	0.0070
106–120	0.1655	0.0095	0.0045	0.0105	106–120	0.2647	0.0078	0.0047	0.0091
120–133	0.2485	0.0169	0.0080	0.0187	120–133	0.3630	0.0141	0.0068	0.0156
133–150	0.2576	0.0210	0.0197	0.0287	133–150	0.4334	0.0179	0.0129	0.0221
150–171	0.2903	0.0259	0.0103	0.0279	150–171	0.4713	0.0230	0.0083	0.0245
171–200	0.3209	0.0315	0.0112	0.0335	171–200	0.4906	0.0276	0.0095	0.0292
200–320	0.3752	0.0286	0.0114	0.0308	200–320	0.5042	0.0244	0.0092	0.0261
320–500	0.4372	0.0655	0.0287	0.0715	320–500	0.5248	0.0610	0.0131	0.0624
500–2000	0.4071	0.1556	0.0824	0.1761	500–2000	0.6878	0.1862	0.0413	0.1907
$2.4<|y |<5$ ($\mathrm{e}\mathrm{e}$ only)									
40–76	−0.3104	0.0912	0.1378	0.1652					
76–86	$-$0.2174	0.0214	0.0210	0.0300					
86–96	0.0635	0.0060	0.0146	0.0158					
96–106	0.2834	0.0183	0.0439	0.0475					
106–120	0.4412	0.0567	0.0696	0.0898					
120–150	0.5972	0.0851	0.0476	0.0975					
150–320	0.8412	0.1567	0.0851	0.1783					

## Systematic uncertainties

The largest experimental uncertainties originate from the background estimation, the electron energy correction, the muon momentum correction, and the unfolding procedure. The dominant contribution to the background uncertainty is the statistical uncertainty in the background data control sample. The theoretical uncertainty of the cross section in the MC background samples also contributes to the systematic uncertainty in the estimation of the background.

After energy corrections to central electrons are applied, we find that there is a 0.4 % offset in the position of the Z peak between data and simulation in the barrel and a 0.5 % offset in the endcaps. This difference is assigned as the systematic uncertainty in the central electron energy calibration.

In order to estimate the uncertainty in the energy calibration of forward electrons, the parametrized function of the correction factor is scaled up and down by its statistical uncertainty. The difference in ${A}_{\mathrm{FB}}$ before and after changing the correction factor is assigned as a systematic uncertainty.

The systematic uncertainty in the muon momentum correction is estimated with a similar approach. The muon momentum correction is scaled up and down by its statistical uncertainty and the difference in ${A}_{\mathrm{FB}}$ resulting from the change of the muon momentum correction is assigned as systematic uncertainty. We find that the contributions of the uncertainties in the efficiency scale factors (trigger, identification, and isolation) and in the pileup reweighting factors to the uncertainty in ${A}_{\mathrm{FB}}$ are small.

For forward HF electrons, the uncertainties in the electron $\eta$ correction and in the global normalization factor contribute to the systematic uncertainty in ${A}_{\mathrm{FB}}$. In addition, the energy calibration varies approximately 5 % between $+\eta$ and $-\eta$. To account for this asymmetric effect in the energy calibration, the ${A}_{\mathrm{FB}}$ distribution is measured using one forward electron in $+\eta$ or $-\eta$, separately, along with one central electron and half of the difference in ${A}_{\mathrm{FB}}$ is assigned as a systematic uncertainty. The systematic uncertainty varies from 0.005 to 0.03 as a function of dielectron invariant mass.

The systematic uncertainty in the unfolding procedure is estimated using a closure test in simulation. Any residual shown in the closure test of the unfolding procedure is assigned as the systematic uncertainty.

The theoretical uncertainties which affect the detector acceptance originate from the uncertainties in PDFs (CT10 [27, 43] and NNPDF 2.0 [44]) and from uncertainties in the FSR modeling [45].

The systematic uncertainty in ${A}_{\mathrm{FB}}$ depends on the mass of the dilepton pair. Table 1 gives the maximum value of this uncertainty from each source, for different regions of *|y|*.

## Results

A comparison of the unfolded, background-subtracted ${A}_{\mathrm{FB}}(M)$ distributions for $\mu \mu$ and $\mathrm{e}\mathrm{e}$ events in the four central rapidity regions is shown in Fig. 3. The statistical and systematic uncertainties are added in quadrature. The measured ${A}_{\mathrm{FB}}(M)$ distributions agree for $\mu \mu$ and $\mathrm{e}\mathrm{e}$ events in all rapidity regions.

The unfolded ${A}_{\mathrm{FB}}(M)$ measurements for $\mu \mu$ and $\mathrm{e}\mathrm{e}$ events, within $|y | < 2.4$, are combined under the assumption that the uncertainties in the muon and electron channels are uncorrelated. Any effect of the correlation between the $\mu \mu$ and $\mathrm{e}\mathrm{e}$ systematic uncertainties in the pileup correction, FSR modeling, and the normalization of MC simulations in the background estimation is found to have a negligible effect on the combination.

Figure 4 shows the combined results for the four central rapidity regions up to 2.4. The combined result is compared with the powheg (NLO) prediction with CT10 PDFs. The effective weak mixing angle, $\sin ^2\theta ^\text {eff}_{\text {lept}}$ = 0.2312, is used for the powheg prediction. For all rapidity regions, the combined ${A}_{\mathrm{FB}}(M)$ values are in a good agreement with the powheg prediction. The uncertainty in the theoretical prediction (powheg) originates from the statistical uncertainty in the MC sample, the uncertainties in the PDFs, and the variations of factorization and renormalization scales (simultaneous variation between values 2*M*, *M*, and *M* / 2, with *M* corresponding to the middle of the invariant mass bin). Table 2 summarizes the combined ${A}_{\mathrm{FB}}$ quantity for each rapidity region.

The unfolded ${A}_{\mathrm{FB}}$ distribution for the forward rapidity region ($2.4< |y | < 5$) is shown in Fig. 5. The forward rapidity region extends the scope of the measurement beyond that of the previous CMS result at $\sqrt{s}=7$ $\,\mathrm{TeV}$. Because ${A}_{\mathrm{FB}}$ in the forward rapidity region is diluted less, the measured ${A}_{\mathrm{FB}}$ quantity is closer to the parton-level asymmetry after the unfolding process, than it is in the central rapidity bins. The unfolded ${A}_{\mathrm{FB}}$ ($M_{\mathrm{e}^{+}\mathrm{e}^{-}}$) for $2.4<|y |<5$ agrees with the powheg predictions.

## Summary

We report a measurement of the forward–backward asymmetry of oppositely charged $\mu \mu$ and $\mathrm{e}\mathrm{e}$ pairs produced via a $\mathrm{Z}/\gamma ^*$ boson exchange at $\sqrt{s} = 8$ $\,\mathrm{TeV}$ with a data sample corresponding to an integrated luminosity of 19.7$\,\mathrm{fb}^{-1}$. The ${A}_{\mathrm{FB}}$ measurement is performed as a function of the dilepton invariant mass between 40$\,\text {GeV}$ and 2$\,\mathrm{TeV}$ for $\mu \mu$ and $\mathrm{e}\mathrm{e}$ events in 4 dilepton rapidity bins up to $|y | = 2.4$. For $\mathrm{e}\mathrm{e}$ events with $2.4< |y | < 5$, the ${A}_{\mathrm{FB}}$ measurement is performed for dielectron masses between 40 and 320$\,\text {GeV}$. The large data sample collected at 8$\,\mathrm{TeV}$ extends the measurement of ${A}_{\mathrm{FB}}$ in the high mass region compared to previous results. The final ${A}_{\mathrm{FB}}$ values are corrected for detector resolution, acceptance, and final state radiation effects. The measurements of ${A}_{\mathrm{FB}}(M)$ are consistent with standard model predictions.
